# Expulsion of Tape Worm by Pumpkin Seeds

**Published:** 1852-09

**Authors:** 


*Expulsion of Tape Worm by Pumpkin Seeds.* Communicated for the Boston Medical and Surgical Journal.

Having recently had an opportunity to administer the remedy for tapeworm recommended in the Journal for October 8, 1851, I take the liberty to send you a brief account of its operation.

The patient, an adult, had taken, several weeks since, by direction of a physician, some extract of male fern followed by castor oil, which expelled about four feet of worm, together with a number of fragments. The remedy was repeated, but no further benefit was obtained.

There being sufficient evidence, however, that the difficulty was not overcome, I determined, as the case fell under my charge, to try the pumpkin seed orgeat, which was prepared and administered as follows: Six ounces of common pumpkin seeds were thoroughly bruized in a mortar, without removing the outer shells, and a sufficient quantity of water was added to afford by straining and expression one pint of liquid. At six o'clock, A. M., the patient took one half of the liquid, or orgeat, and in two hours after half an ounce of castor oil. A slight movement of the bowels followed, with a few fragments of the worm. At ten o'clock, half an ounce more of oil was given, the abdomen was rubbed with sulph. ether, and cold water was directed to be used freely. No food to be taken until after the operation. At twelve o'clock the bowels were evacuated, and an entire worm discharged, eight feet and seven inches in length.

Although the patient is quite feeble from the effects of pulmonary and hepatic disease, no inconvenience has resulted from the remedy.

ROCHESTER, N. Y., *July* 13, 1852. W. W. ELY.

